# Retraction Note: MiRNA-211 suppresses cell proliferation, migration and invasion by targeting SPARC in human hepatocellular carcinoma

**DOI:** 10.1038/s41598-023-39043-3

**Published:** 2023-07-24

**Authors:** Biao Deng, Lei Qu, Jinfang Li, Jiaqing Fang, Shouwen Yang, Zhongwei Cao, Zhechuan Mei, Xing Sun

**Affiliations:** 1 Department of General Surgery, Shanghai First People’s Hospital, Shanghai Jiao Tong University, 100 Haining Road, Shanghai, 200080 China; 2 Department of Neurology, The Second Affiliated Hospital, Chongqing Medical University, Chongqing, China; 3 Department of Gastroenterology, Tianyou Hospital, TongJi University, 500 Zhennan Road, Shanghai, 200331 China; 4 Department of Gastroenterology, Shanghai First People’s Hospital, Shanghai Jiao Tong University, 100 Haining Road, Shanghai, 200080 China; 5 Department of Gastroenterology, The Second Affiliated Hospital, Chongqing Medical University, Chongqing, China

Retraction of: *Scientific Reports* 10.1038/srep26679, published online 27 May 2016

The Editors have retracted this Article.

After the publication of this Article it was brought to the Editors' attention that it shows multiple instances of overlap of data between various figures, where the data are described as corresponding to different samples or conditions. Additionally, non-inclusive overlaps with differently described data were identified in subsequently published work^1^. The Authors were contacted and asked for an explanation and for the raw data but have not responded. The Editors therefore no longer have confidence in the data reported in this Article.

Biao Deng, Lei Qu, Jiaqing Fang, Shouwen Yang, Zhongwei Cao, Zhechuan Mei & Xing Sun did not respond to the correspondence about the retraction. The Editors were not able to establish the current contact details for Jinfang Li.

## References

[1] Ma Y-S, Chu K-J, Ling C-C, Wu T-M, Zhu X-C, Liu J-B (2020). RETRACTED: Long noncoding RNA OIP5-AS1 promotes the progression of liver hepatocellular carcinoma via regulating the hsa-miR-26a-3p/EPHA2 axis. Mol. Ther. Nucleic Acids.

